# Association Between Sleep Apnea Symptoms Subtypes and Obesity

**DOI:** 10.3390/jcm15103969

**Published:** 2026-05-21

**Authors:** Mario Henríquez-Beltrán, Daniel Solomons, María F. Troncoso, Montserrat Sánchez Martínez, Jorge Jorquera, Gonzalo Labarca

**Affiliations:** 1 Núcleo de Investigación en Ciencias de la Salud, Universidad Adventista de Chile, Chillán 3780000, Chile; eliashbm@hotmail.com; 2 Department of Respiratory Diseases, School of Medicine, Pontificia Universidad Católica de Chile, Santiago 8330024, Chile; 3 Translational Research in Respiratory Medicine, Hospital Universitari Arnau de Vilanova-Santa Maria, Biomedical Research Institute of Lleida (IRBLleida), 25198 Lleida, Spain; 4 Escuela de Kinesiología, Facultad de Ciencias de la Salud, Universidad Católica Silva Henríquez, Santiago 8320000, Chile; dsolomons@ucsh.cl; 5 Instituto de Matemática, Física y Estadística, Universidad de Las Américas, Sede La Florida, Av. Walker Martínez 1360, Santiago 8240000, Chile; 6 CIBERES, Pulmonology IIS Fundación Jiménez Díaz Madrid, 28040 Madrid, Spain; mftroncoso@quironsalud.es; 7 Grupo de Estudio Trastornos Respiratorios del Sueño, Clínica Las Condes, Santiago 7591047, Chile; msanchezm@clinicalascondes.cl (M.S.M.); jjorquera@clinicalascondes.cl (J.J.); 8 Department of Respiratory, Allergy and Sleep Medicine, Mayo Clinic, Jacksonville, FL 32224, USA

**Keywords:** obstructive sleep apnea, OSA, obesity, excessive daytime sleepiness, sleep

## Abstract

**Background and Objectives**: Obstructive sleep apnea (OSA) is a heterogeneous disease with diverse clinical presentations and high global prevalence. Obesity is a common comorbidity in OSA, but its relationship with symptom subtypes remains unclear. This study aimed to evaluate the association between OSA symptom subtypes and obesity in a clinical cohort. **Methods**: This observational study analyzed data from the Santiago Obstructive Sleep Apnea (SantOSA) prospective clinical cohort, including adults with OSA confirmed by home sleep apnea testing. Symptom subtypes were identified using latent class analysis. Associations between obesity and symptom subtypes were evaluated using multivariable regression models adjusted for age, sex, tobacco use, RDI, T90%, TST, hypertension, and diabetes. Statistical significance was set at *p* < 0.05. **Results**: A total of 1167 patients were included (943 men). Latent class analysis identified three symptom subtypes: non-sleepy, disturbed sleep, and excessive daytime sleepiness. Among obese patients, 30.7%, 50.0%, and 19.3% were classified into these subtypes, respectively. Obesity prevalence was 50.2%, and compared with non-obese OSA patients, obese patients showed a higher prevalence of severe OSA (46.1% vs. 26.9%), hypertension (54.4% vs. 34.9%), and diabetes (37.2% vs. 19.4%), as well as higher ESS scores and higher RDI and T90% values (all *p* < 0.01). In adjusted analyses, obesity remained independently associated with the excessive daytime sleepiness subtype after controlling for age, sex, tobacco use, RDI, T90%, TST, and comorbidities. **Conclusions**: Obesity is highly prevalent in OSA and is associated with specific symptom-defined phenotypes, particularly excessive daytime sleepiness and disturbed sleep. These findings support the relevance of considering symptom profiles alongside traditional severity metrics.

## 1. Introduction

Obstructive sleep apnea (OSA) is a highly prevalent and clinically diverse disorder, affecting an estimated one billion individuals worldwide, with nearly 45% presenting moderate-to-severe forms [1,2]. Beyond its diagnosis through the apnea-hypopnea index (AHI), OSA is increasingly recognized as a multifaceted disease with diverse symptom profiles and substantial clinical variability.

In recent years, different approaches have been proposed to redefine OSA according to specific phenotypes, with some focusing on the severity of sleepiness-related symptoms. To better characterize individual patients with OSA, recent studies have emphasized the identification of disease subtypes, underscoring the importance of tailored clinical assessment [3,4,5,6,7].

This heterogeneity is clinically relevant, as OSA frequently coexists with cardiometabolic comorbidities that contribute to adverse health outcomes [6,7,8,9,10].

In this context, obesity is one of the most common comorbidities among patients with OSA [11,12]. However, its prevalence within OSA populations shows considerable variability depending on disease severity and population characteristics. A recent large-scale analysis including the Sleep Heart Health Study (SHHS) and other community-based cohorts reported that the prevalence of obesity among individuals with OSA is approximately 31–33%, increasing progressively with disease severity to around 39.5% in moderate-to-severe OSA and up to 47% in severe OSA. These findings highlight that, despite the well-established relationship between obesity and OSA, a substantial proportion of patients with OSA do not have obesity. This reinforces the concept of OSA as a heterogeneous condition with multiple phenotypic expressions beyond body weight alone, supporting the need to consider clinical subtypes in addition to traditional severity metrics [13].

Strong evidence suggests that OSA patients with excessive daytime sleepiness are at a higher risk of experiencing injurious outcomes [14,15]. While there are multiple methods to determine the sleepy phenotype, one of the most validated is the cluster analysis, initially proposed by Ye et al. [16], and later validated by the Sleep Apnea Global Interdisciplinary Consortium (SAGIC), Canada, and Chile [17]. This unsupervised cluster analysis is based on symptom subtypes derived from sleep questionnaires and can identify between 3 and 5 clusters. Generally, the excessive sleepiness cluster is associated with worse cardiovascular outcomes, beyond the AHI [3]. An alternative approach is based on the categorical analysis of the Epworth Sleepiness Scale (ESS), whereby populations exhibiting sleepiness are identified based on scores exceeding 10 points [16].

Obesity constitutes a significant contributing factor to OSA [18]; however, there is no clear evidence regarding the association between Obese OSA and symptom subtypes. This study aimed to determine the association between obesity and sleep apnea symptoms in a clinic-based cohort.

## 2. Materials and Methods

### 2.1. Study Design and Participants

An observational study was conducted in accordance with the current recommendations outlined in the Strengthening the Reporting of Observational Studies in Epidemiology (STROBE) statement. [19], employing data from the Santiago Obstructive Sleep Apnea (SantOSA) study.

### 2.2. Cohort Description

SantOSA is a prospective, clinic-based registry/cohort of adult patients referred for clinical evaluation of obstructive sleep apnea at a tertiary center located in the metropolitan region of Santiago, Chile. The SantOSA protocol is registered in the ISRCTN registry (ISRCTN62293645). The research registry protocol was approved by the Institutional Review Board of Clínica Las Condes, Santiago, Chile (approval number: 00008758; approval date: 9 March 2018). The present manuscript corresponds to a secondary observational analysis of SantOSA registry/cohort data from adults evaluated between 2009 and 2020. Written informed consent was obtained from all participants according to the approved registry procedures. The key inclusion criteria were: (i) adults aged > 18 years, and (ii) individuals referred for the sleep study due to clinical suspicion of OSA, based on symptoms such as snoring, witnessed apneas, excessive daytime sleepiness, or the presence of major cardiovascular comorbidities. Key exclusion criteria were: (i) participants with other sleep disorders, including periodic limb movements of sleep (PLMS), NREM-related arousal disorders, sleep-related hypoxemia not clearly attributable to obstructive sleep apnea, or poor sleep due to alternative etiologies, and (ii) those who declined to provide written informed consent.

In the present study, 305 records were excluded due to non-OSA status (RDI < 5 events/h), and 76 were excluded due to incomplete data. After applying the inclusion and exclusion criteria, 1167 participants were retained for analysis. Only the first valid HSAT was considered for individuals with repeated recordings, and all included patients had a confirmed diagnosis of OSA (RDI ≥ 5 events/h) and complete clinical and sleep-related data. While no formal power calculation was performed, the large cohort and observational design provided sufficient representation to address the primary study objectives.

All participants underwent standardized clinical evaluations that assessed sociodemographic characteristics, lifestyle habits (such as tobacco and alcohol use), comorbidities including hypertension (HTN), diabetes mellitus (DM), coronary heart disease (CHD), and chronic obstructive pulmonary disease (COPD), as well as medication use. These comorbidities were included in the descriptive analysis and incorporated into the multivariable regression and cluster analyses to evaluate their association with OSA symptom subtypes.

Anthropometric measurements included weight and height, which were recorded following an overnight fast and while wearing only underwear. The body mass index (BMI; kg/m^2^) was calculated, and neck circumference was measured using a flexible tape measure at the level of the cricoid.

### 2.3. Sleep Assessment

At baseline, participants completed a self-reported sleep symptom questionnaire, which was specifically designed by the authors to assess sleep patterns, level of daytime sleepiness, snoring intensity, observed apneas, insomnia, episodes of nocturnal suffocation, and morning headaches. This instrument has been used in previous peer-reviewed studies, showing consistent application across clinical cohorts [4,5,6,7]. Additionally, the Spanish version of the Epworth Sleepiness Scale (ESS), validated by Chiner et al. (1999), was used in this study for academic research purposes [20]. This version was published with full methodological details and the complete questionnaire included as an annex. No specific restrictions regarding academic use are indicated in that publication [20]. Additionally, total sleep time (TST) was defined as self-reported habitual sleep duration, expressed in hours per night.

To conduct the HSAT, we used a validated type-3 sleep test (using the Embletta Multi-Parameter Recorder, Natus Medical, Inc., Middleton, WI, USA), including nasal pressure assessment (measuring airflow), thoracic and abdominal inductance plethysmography, body position assessment, audio assessment via a microphone, and pulse oximetry. The HSAT device demonstrated a 92.4% sensitivity and 85.7% specificity in the quantification of RDI when evaluated in a comparative analysis with polysomnography (PSG) in patients with suspected OSA syndrome [21]. The HSAT was performed once at the beginning of the study. The HSAT analysis was conducted manually in accordance with prevailing guidelines by a respiratory disease specialist (JJ), who was unaware of the clinical histories of the participants [22,23]. The following variables were included in the HSAT analysis: (i) Respiratory Disturbance Index (RDI) was defined, in the context of this type 3 home sleep apnea test, as the number of apneas and hypopneas associated with a ≥3% oxygen desaturation per hour of recording. Because electroencephalography was not available, respiratory effort-related arousals (RERAs) were not included in this index; (ii) mean oxygen saturation (SpO_2_); (iii) minimum SpO_2_; (iv) total time with oxyhemoglobin saturation below 90% (T90%); and (v) oxygen desaturation index (ODI) at 3%. OSA was classified as RDI ≥ 5 events/h, mild OSA as RDI between 5 and 15 events/h, moderate OSA as RDI between 15 and 30 events/h, and severe OSA as RDI ≥ 30 events/h.

### 2.4. Exposure Definition

Obesity was defined as greater than or equal to 30 kg/m^2^, which was further subdivided into three categories: grade I (BMI, 30–34.9 kg/m^2^), grade II (BMI, 35–39.9 kg/m^2^), and grade III (BMI, equal to or greater than 40.0 kg/m^2^) [24].

### 2.5. Latent Class Analysis for Symptom Subtype Identification

**Figure 1:**
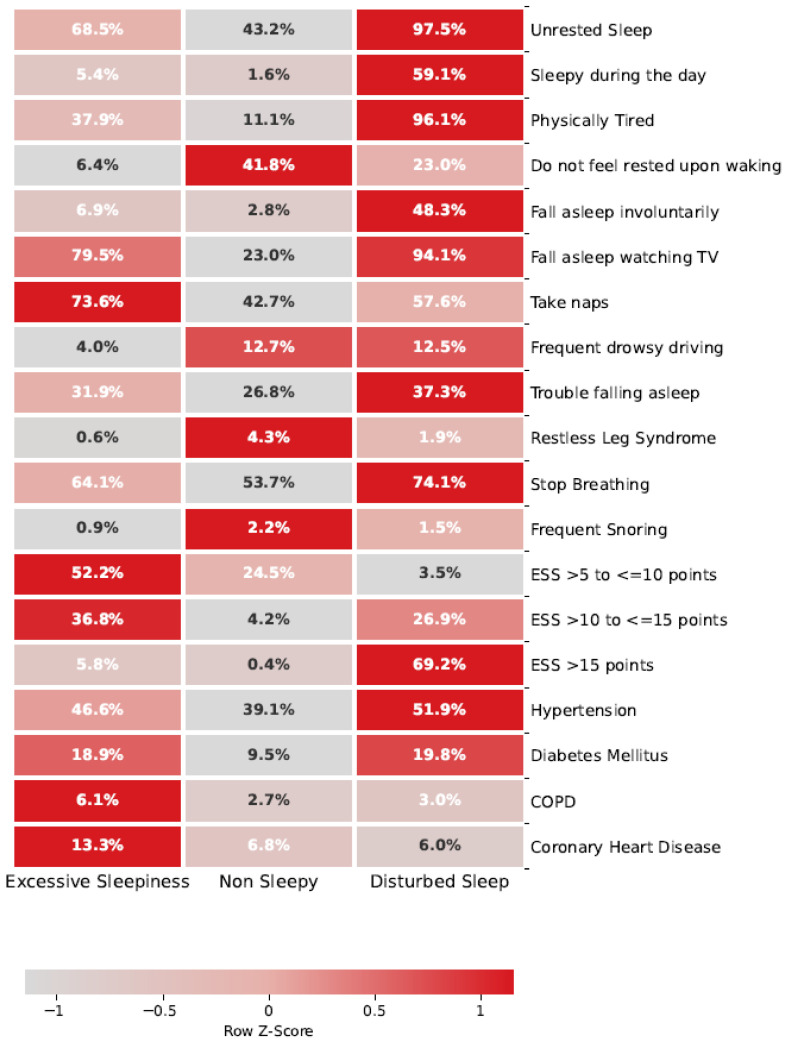
The heat map presents the prevalence of symptoms and comorbidities in three patient subgroups: Excessive Sleepiness, Non-Sleepy, and Disturbed Sleep. The percentages within each cell represent the proportion of patients within the respective subgroup reporting the corresponding symptoms or condition. The color gradient (Z-scores) reflects the relative prevalence of each variable within the subgroups, with darker red tones indicating higher values and lighter tones representing lower values. This visualization highlights patterns of symptom clustering and comorbidity distribution, facilitating the identification of trends across subgroups.

We conducted a latent class analysis (LCA) to identify OSA symptom subtypes, following the methodology previously described by Mazzotti et al. [25]. To reproduce the original clustering procedure, we included the same 15 sleep-related questions from SHHS visit 1. Fourteen questions were dichotomized (yes/no), and the Epworth Sleepiness Scale (ESS) was categorized as 0–5, 6–10, 11–15, and >15. In addition, four comorbidities, hypertension, diabetes mellitus, chronic obstructive pulmonary disease, and coronary heart disease, were incorporated, following previous publications [3]. The summary of the question and the heat map are presented in Figure 1. The optimal number of clusters was determined using the Bayesian Information Criterion (BIC), selecting the most parsimonious solution. Based on this procedure, three clusters were retained:

1. Non-Sleepy: characterized by a lower overall probability of sleep-related complaints and daytime sleepiness-related symptoms compared with the other subtypes.
2. Disturbed Sleep: characterized by a higher probability of sleep disturbance and non-restorative sleep symptoms, including not feeling rested upon waking, trouble falling asleep, and related sleep complaints.
3. Excessive Daytime Sleepiness (EDS): characterized by a higher probability of daytime sleepiness-related symptoms, including sleepiness during the day, involuntary sleep episodes, physical tiredness, napping, and falling asleep while watching TV, as illustrated in Figure 1.

### 2.6. Data Analysis

Baseline characteristics were analyzed using median [interquartile range, IQR] for continuous variables, while dichotomous variables were expressed as percentages (%). To determine the clinical differences between obese and non-obese OSA, we performed chi-square and parametric tests.

The association between symptom subtypes and obesity was determined using two additional analyses. Firstly, we determined the association between symptom subtypes and BMI using a linear regression model adjusting for sex, age, hypertension, diabetes, tobacco, RDI, T90%, and total sleep time (TST), with values expressed in a beta coefficient (95% confidence interval). In a second analysis, we determined the association between symptom subtypes with obesity using a multivariable regression model, adjusted by the same covariates described above. Values were expressed as adjusted odd ratios (aOR) with the corresponding 95% CI. Total sleep time (TST) was included as a covariate to account for differences in habitual sleep duration across participants.

In a sensitivity analysis, we tested for interactions between symptom subtypes and age, sex, and OSA severity. Finally, we conducted a Spearman correlation between BMI and both RDI and ESS. A *p*-value < 0.05 was considered significant and all analyses were performed using R software Version 4.0.2 (R Core Team; Vienna, Austria) and Graph Pad Prism Version 10.2.3.

## 3. Results

**Figure 2:**
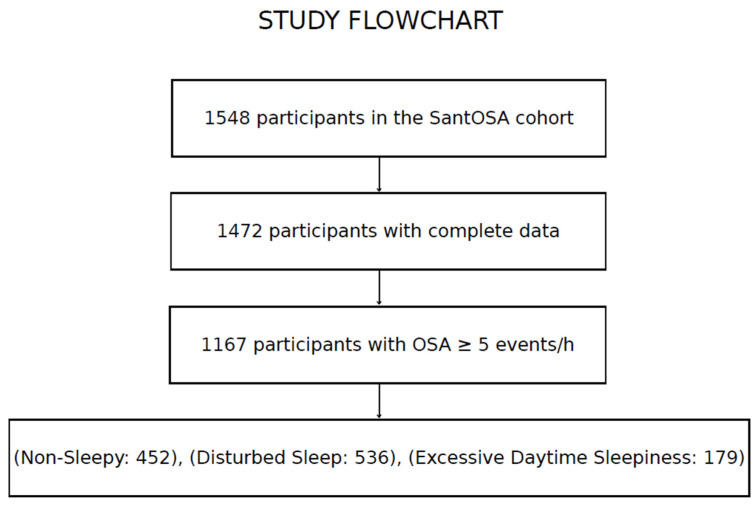
Participant selection from the SantOSA cohort. Of 1548 participants initially included, 1472 had complete data. After excluding participants without obstructive sleep apnea, defined as respiratory disturbance index < 5 events/h, 1167 participants with obstructive sleep apnea were retained for analysis. The final analytic sample included 452 Non-Sleepy, 536 Disturbed Sleep, and 179 Excessive Daytime Sleepiness participants.

A total of 1167 participants were included in this study, comprising 943 males and 224 females. The study flowchart is presented in Figure 2 (see below).

Latent class analysis identified three symptom-based OSA clusters: Non-Sleepy, Disturbed Sleep, and Excessive Daytime Sleepiness. The distribution of participants across clusters and the specific symptom profiles are illustrated in Figure 1.

The prevalence of obesity was 586/1167 (50.2%). Among obese OSA patients, 23%, 30.9%, and 46% were mild, moderate, and severe OSA, respectively. Among obese patients, Non-sleepy was 30.7%, Disturbed sleep was 50%, and excessive daytime sleepiness was 19.3%. Compared with non-obese OSA patients, Obese patients showed higher ESS, with a median of 9 [6–13.8] points, more females (23.2%), and a higher prevalence of hypertension and diabetes, as well as higher RDI, T90%, and total sleep time (TST). Table 1 shows clinical differences between groups.

### Association Between BMI and Symptom Subtypes

**Figure 3:**
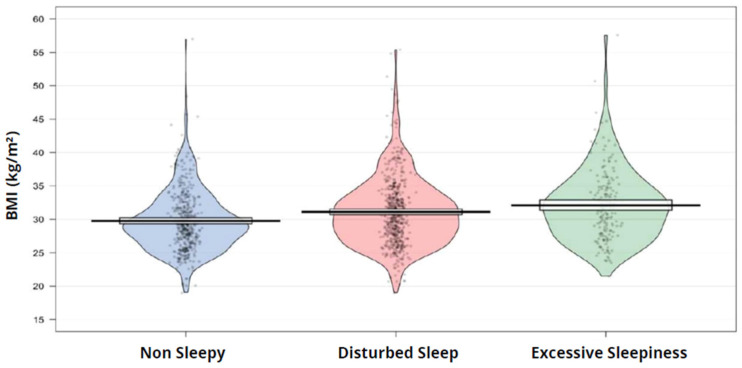
The plot shows the distribution of BMI values, individual observations, and summary estimates across the Non-sleepy, Disturbed sleep, and Excessive daytime sleepiness subtypes.

**Figure 4:**
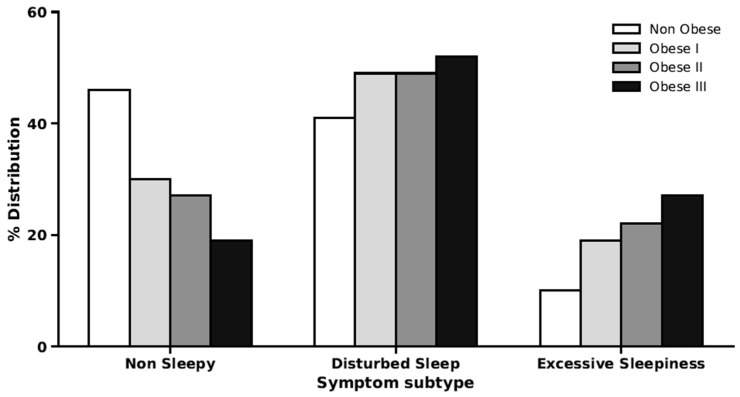
The figure shows the percentage of participants classified as Non-sleepy, Disturbed sleep, or Excessive daytime sleepiness within obesity grade I (BMI 30–34.9 kg/m^2^), grade II (BMI 35–39.9 kg/m^2^), and grade III (BMI ≥ 40 kg/m^2^).

The association between BMI and symptom subtypes and the association within the obesity category is shown in Figure 3 and Figure 4**,** respectively. In the subsequent analysis, we found a weak correlation between BMI and both ESS and RDI. The Spearman’s Rho for ESS was 0.16, and for RDI it was 0.35. The adjusted linear regression model showed a significant beta coefficient between disturbed sleep and excessive daytime sleepiness subtype with BMI, the beta coefficient (95% CI) was 0.89 (0.30–1.48), and 1.36 (0.54–2.19), respectively. Table 2 shows the Linear regression model (total), determining BMI changes with different symptom subtypes.

Finally, the risk of disturbed sleep and excessive sleepiness was higher in the obese group, the adjusted OR for this association was 1.62 (1.23–2.13, *p* < 0.01) and 2.02 (1.37–2.99, *p* < 0.01), respectively. Table 3 shows the multivariable regression model, determining the risk of obesity with symptoms subtypes. In the sensitivity analysis, we found no interaction between symptom subtypes and age, sex, and OSA severity.

## 4. Discussion

The main findings of this study are the following: (1) In our clinic-based cohort, the prevalence of obese OSA was 50%, and regarding OSA severity, 46% of the obese population reported severe OSA; (2) Obesity increased the risk of both disturbed sleep and excessive daytime sleepiness subtypes, and (3) the association between obesity and BMI with excessive daytime sleepiness subtype is not influenced by age, sex, tobacco use, RDI, T90%, or other comorbidities.

In this study, we hypothesize that symptom subtypes will show a higher prevalence in the obese OSA group regardless of OSA severity, and therefore, will be an independent marker of disease severity beyond the RDI. Accordingly, we modeled obesity as the dependent variable in order to evaluate its distribution across the symptom subtypes. This analytic approach is consistent with previous clustering studies [25], where comorbidities and anthropometric measures were examined as outcomes associated with symptom-defined phenotypes, rather than as exposures.

As a result, we reported prevalence and clinical characteristics similar to previous reports, including a recent analysis of the SHHS. One-half of our sample reported obesity, and this group also reported clinical differences compared with non-obese OSA. As expected, this group had an increased risk of comorbidities, more severe OSA, and were more likely to be of a more advanced age.

Beyond confirming the well-established association between obesity and OSA severity, our findings demonstrate that obesity is also linked to specific symptom-defined subtypes, particularly excessive daytime sleepiness and disturbed sleep. This suggests that obesity may serve as an independent clinical marker of symptom-defined phenotypes, reinforcing the relevance of considering symptom profiles in addition to conventional indices of disease severity such as the RDI.

In this scenario, the association between obesity and OSA reflects a complex interplay of pathophysiological mechanisms that differ by sex. Evidence from the SNOOzzE study indicates that, in men, obesity contributes to a higher apnea-hypopnea index (AHI) through increased upper airway collapsibility, reduced circulatory delay, and an elevated arousal threshold [26]. In women, the relationship is primarily driven by upper airway collapsibility and circulatory delay, leading to shorter but more frequent respiratory events. Moreover, the balance between apneas and hypopneas is clinically relevant, as apneas generate greater intrathoracic pressure swings, amplifying cardiovascular strain and overall disease severity. These distinctions emphasize the importance of considering sex-specific mechanisms and event type when evaluating the clinical impact of OSA.

In addition, our findings indicated that obesity is associated with an elevated risk of both disturbed sleep and excessive daytime sleepiness. OSA is approximately 4 times as common in individuals with obesity compared to individuals who are not overweight or obese [27].

While many patients with OSA may be asymptomatic or minimally symptomatic, excessive daytime sleepiness is the most frequently reported symptom among symptomatic individuals. Additionally, other symptoms such as gastroesophageal reflux disease and poor mental health, which share a reciprocal relationship, have also been associated with OSA and in turn associated with disturbed sleep.

Several pathophysiological mechanisms have been proposed to explain the association between obesity and OSA, with increased upper airway collapsibility representing the central pathway. In this context, fat deposition in the parapharyngeal region may directly reduce upper airway caliber, increasing its susceptibility to collapse during sleep. In addition, abdominal adiposity decreases lung volumes and reduces caudal traction on the upper airway, further promoting airway instability [28].

These effects are complemented by fat accumulation in the tongue, which has been associated with greater upper airway obstruction and a higher burden of respiratory events [29]. Importantly, these mechanisms may contribute to sleep fragmentation and intermittent hypoxia, which are key processes underlying excessive daytime sleepiness and other symptom manifestations. Together, this framework supports a mechanistic link between obesity, OSA severity, and the clinical expression of symptom-based subtypes.

Recent evidence indicates that OSA is also common in individuals without obesity [18], highlighting that factors beyond body weight contribute to disease development and symptom expression. This reinforces the need to consider obesity as one, but not the only, driver of symptom-defined phenotypes.

Although weight loss may not always normalize OSA due to inter-patient variability, addressing obesity remains crucial, not only for OSA management but also for reducing cardiovascular risk and improving mental health and quality of life. Accordingly, weight management should be considered a key component of a comprehensive treatment plan for patients with OSA, particularly those who are overweight or obese, complementing other therapeutic interventions. These considerations are especially relevant given our findings that obesity is associated with specific symptom subtypes, highlighting the need for personalized approaches beyond conventional severity indices [27].

In contrast, previous results have shown that excessive sleepiness was independently associated with cardiovascular mortality across the sleep-related symptoms [3]. However, these results were only performed among patients with moderate to severe OSA, in contrast to our results, where we included mild patients.

Considering the limitations of the study, it is noteworthy that the data (SantOSA) included in this analysis were obtained from a single center located in an urban area of Santiago, Chile, where patients had a high income and educational level. In addition, the use of respiratory polygraphy may have led to the underdiagnosis of respiratory events in our cohort. The calculation of metrics such as hypoxic exposure was not possible due to the lack of signal data and appropriate analytical tools. The inclusion of these variables would have provided a more complete characterization of sleep-disordered breathing and its physiological consequences, thereby enriching the results of the study. Another limitation is that the sleep symptom questionnaire used in this study was specifically designed by the investigators and, although it has been consistently applied in previous peer-reviewed publications, it has not undergone a formal validation process. This lack of validation may limit the external generalizability of symptom assessment.

Finally, while no formal power calculation was performed to estimate the sample size, the study’s robust cohort and observational design ensured sufficient representation for addressing the primary objectives. Nonetheless, the design does not allow causality to be established, as this was beyond the scope and objectives of our study.

Despite these limitations, several strengths of this study should be highlighted. First, this analysis was conducted in a well-characterized clinical cohort with standardized sleep assessments, ensuring consistency in data acquisition. Second, the use of latent class analysis enabled the identification of clinically meaningful symptom-defined subtypes, providing a more refined characterization of OSA heterogeneity beyond traditional severity metrics. Finally, the integration of clinical variables with objective sleep parameters strengthens the internal consistency of the findings and supports their clinical relevance.

Building on these strengths, our findings provide relevant clinical insights. From a clinical perspective, these results suggest the incorporation of symptom-based phenotyping into the routine evaluation of patients with OSA. Traditional severity metrics, such as the RDI, may not fully capture the heterogeneity of clinical presentation, particularly in relation to obesity. Identifying distinct symptom profiles, including excessive daytime sleepiness and disturbed sleep, may contribute to a more comprehensive assessment of patient burden and support more individualized management strategies. Future studies should determine whether these symptom-defined subtypes are associated with differential treatment responses and long-term clinical outcomes.

## 5. Conclusions

Overall, obesity is associated with excessive daytime sleepiness and disturbed sleep symptom subtypes and represents a highly prevalent comorbid condition among individuals with OSA. Beyond its association with greater disease severity, our findings demonstrate that obesity is consistently linked to specific symptom-defined subtypes, particularly excessive daytime sleepiness and disturbed sleep. Importantly, the association between obesity-related variables, such as body mass index, and these symptom subtypes remained unchanged after adjustment for sociodemographic factors and HSAT-derived measures, suggesting that obesity may act as an independent marker of clinical phenotype rather than solely reflecting disease severity. These findings support the integration of symptom profiles into the clinical assessment of OSA, enabling a more comprehensive characterization of patients beyond conventional indices of disease severity.

## Figures and Tables

**Table 1:** Baseline characteristics (*n* = 1167).

	Non-Obese (*n* = 581)	Obese (*n* = 586)	*p*-Value
	*n* (%) or Median [p25; p75]		
Symptom subtype			<0.01
Non-Sleepy	272 (46.8%)	180 (30.7%)	
Disturbed Sleep	243 (41.8%)	293 (50.0%)	
Excessive Daytime Sleepiness	66 (11.4%)	113 (19.3%)	
Sociodemographic data			
Age, years	53 [42–62]	55 [44–65]	<0.01
Tobacco			
Never smoker	273 (46.9%)	280 (47.8%)	
Former smoker	184 (31.6%)	169 (28.9%)	
Current smoker	124 (21.3%)	137 (23.3%)	
Alcohol			<0.01
Never	135 (23.2%)	180 (30.7%)	
Occasional	402 (69.2%)	352 (60.0%)	
Frequent	44 (7.57%)	54 (9.2%)	
Sex			<0.01
Male	493 (84.9%)	450 (76.8%)	
Female	88 (15.1%)	136 (23.2%)	
Comorbidities			
Hypertension	203(34.9%)	319 (54.4%)	<0.01
Diabetes	113 (19.4%)	218 (37.2%)	<0.01
COPD	19 (3.27%)	23 (3.9%)	
CHD	38 (6.54%)	56(9.7%)	<0.05
Sleep variables			
RDI	18.1 [10.8–31.2]	27.5 [15.8–44.8]	<0.01
T90%	2.6 [0.5–10.3]	9.45 [ 2.0–34.1]	<0.01
TST, hrs.	6.5 [ 5.5–7.0]	7.0 [6.0–8.0]	0.04
ESS, point	8.0 [5.0–11.0]	9.0 [6.0–13.8]	<0.01
OSA severity			<0.01
Mild	232 (39.9%)	135 (23.0%)	
Moderate	193 (33.2%)	181 (30.9%)	
Severe	156 (26.9%)	270 (46.1%)	

Abbreviations: number, *n*; percentile, *p*; respiratory disturbance index, RDI; the total time with oxyhemoglobin saturation lower than 90%, T90%; total sleep time, TST; Epworth Sleepiness scale, ESS; obstructive sleep apnea, OSA; chronic obstructive pulmonary disease, COPD; coronary heart disease, CHD.

**Table 2:** Linear regression model (total), determining BMI changes with different symptom subtypes.

	Model 1 (Beta, 95% CI)	Model 2 (Beta, 95% CI)	Model 3 (Beta, 95% CI)
Non-Sleepy	Reference category	Reference category	Reference category
Disturbed Sleep	1.36 (0.74–1.99), *p* < 0.01	1.38 (0.76–2.01), *p* < 0.01	0.96 (0.38–1.55), *p* < 0.01
Excessive daytime sleepiness	2.34 (1.47–3.20), *p* < 0.01	2.30 (1.43–3.16), *p* < 0.01	1.49 (0.67–2.32), *p* < 0.01
Adjusted R^2^	0.03	0.04	0.17
Model 1: Non-Adjusted.			
Model 2: Adjusted by Age, Sex, Tobacco, Hypertension, and Diabetes.			
Model 3: Model 2 + RDI + T90% + TST.			

Abbreviation: Body Mass Index, BMI; confidence interval, CI; respiratory disturbance index, RDI; the total time with oxyhemoglobin saturation lower than 90%, T90%; total sleep time, TST.

**Table 3:** Multivariable regression model, determining the risk of obesity with symptoms subtypes.

	Model 1 (OR, 95% CI)	Model 2 (OR, 95% CI)	Model 3 (OR, 95% CI)
Non-Sleepy	Reference category	Reference category	Reference category
Disturbed Sleep	1.82 (1.41–2.35), *p* < 0.01	1.84 (1.43–2.38), *p* < 0.01	1.66 (1.27–2.18), *p* < 0.01
Excessive daytime sleepiness	2.59 (1.82–3.71), *p* < 0.01	1.81 (1.43–3.73), *p* < 0.01	1.49 (1.47–3.17), *p* < 0.01
Model 1: Non-Adjusted.			
Model 2: Adjusted by Age, Sex, Tobacco, Hypertension, and Diabetes.			
Model 3: Model 2 + RDI + T90% + TST.			

Abbreviation: Odds Ratio, OR; confidence interval, CI; respiratory disturbance index, RDI; the total time with oxyhemoglobin saturation lower than 90%, T90%; total sleep time, TST.

## Data Availability

The datasets generated and analyzed during the current study are available from the corresponding author upon reasonable request due to privacy and ethical restrictions.

## References

[1] Benjafield A.V., Ayas N.T., Eastwood P.R., Heinzer R., Ip M.S.M., Morrell M.J., Nunez C.M., Patel S.R., Penzel T., Pépin J.-L. (2019). Estimation of the global prevalence and burden of obstructive sleep apnoea: A literature-based analysis. Lancet Respir. Med..

[2] Lyons M.M., Bhatt N.Y., Pack A.I., Magalang U.J. (2020). Global burden of sleep-disordered breathing and its implications. Respirology.

[3] Labarca G., Dreyse J., Salas C., Letelier F., Jorquera J. (2021). A Validation Study of Four Different Cluster Analyses of OSA and the Incidence of Cardiovascular Mortality in a Hispanic Population. Chest.

[4] Labarca G., Dreyse J., Salas C., Letelier F., Schmidt A., Rivera F., Jorquera J. (2020). Clinical utility of oximetric parameters to identify a high-risk phenotype of moderate-severe Obstructive Sleep Apnea (OSA). Clin. Respir. J..

[5] Labarca G., Dreyse J., Salas C., Schmidt A., Rivera F., Letelier F., Jorquera J. (2021). Risk of mortality among patients with moderate to severe obstructive sleep apnea and diabetes mellitus: Results from the SantOSA cohort. Sleep Breath..

[6] Henríquez-Beltrán M., Dreyse J., Jorquera J., Jorquera-Diaz J., Salas C., Fernandez-Bussy I., Labarca G. (2023). The U-Shaped Association between Sleep Duration, All-Cause Mortality and Cardiovascular Risk in a Hispanic/Latino Clinically Based Cohort. J. Clin. Med..

[7] Jorquera J., Dreyse J., Salas C., Letelier F., Weissglas B., Del-Río J., Henríquez-Beltrán M., Labarca G., Jorquera-Díaz J. (2023). Clinical Application of the Multicomponent Grading System for Sleep Apnea Classification and Incident Cardiovascular Mortality. Sleep Sci..

[8] Henríquez-Beltrán M., Dreyse J., Jorquera J., Weissglas B., Del Rio J., Cendoya M., Jorquera-Diaz J., Salas C., Fernandez-Bussy I., Labarca G. (2024). Is the time below 90% of SpO_2_ during sleep (T90%) a metric of good health? A longitudinal analysis of two cohorts. Sleep Breath..

[9] Zapater A., Gracia-Lavedan E., Torres G., Mínguez O., Pascual L., Cortijo A., Martínez D., Benítez I.D., De Batlle J., Henríquez-Beltrán M. (2023). Proteomic profiling for prediction of recurrent cardiovascular event in patients with acute coronary syndrome and obstructive sleep apnea: A post-hoc analysis from the ISAACC study. Biomed. Pharmacother..

[10] Schmickl C.N., Orr J.E., Sands S.A., Alex R.M., Azarbarzin A., McGinnis L., White S., Mazzotti D.R., Nokes B., Owens R.L. (2024). Loop Gain as a Predictor of Blood Pressure Response in Patients Treated for Obstructive Sleep Apnea: Secondary Analysis of a Clinical Trial. Ann. Am. Thorac. Soc..

[11] Testelmans D., Spruit M.A., Vrijsen B., Sastry M., Belge C., Kalkanis A., Gaffron S., Wouters E.F.M., Buyse B. (2022). Comorbidity clusters in patients with moderate-to-severe OSA. Sleep Breath..

[12] Peppard P.E., Young T., Palta M., Dempsey J., Skatrud J. (2000). Longitudinal study of moderate weight change and sleep-disordered breathing. JAMA.

[13] Esmaeili N., Gell L., Imler T., Hajipour M., Taranto-Montemurro L., Messineo L., Stone K.L., Sands S.A., Ayas N., Yee J. (2025). The relationship between obesity and obstructive sleep apnea in four community-based cohorts: An individual participant data meta-analysis of 12,860 adults. eClinicalMedicine.

[14] Labarca G., Montenegro R., Oscullo G., Henriquez-Beltran M., Uribe J.P., Gómez-Olivas J.D., Garcia-Ortega A., Martínez-García M.Á. (2023). Placebo response in objective and subjective measures of hypersomnia in randomized clinical trials on obstructive sleep apnea. A systematic review and meta-analysis. Sleep Med. Rev..

[15] Lal C., Weaver T.E., Bae C.J., Strohl K.P. (2021). Excessive Daytime Sleepiness in Obstructive Sleep Apnea. Mechanisms and Clinical Management. Ann. Am. Thorac. Soc..

[16] Ye L., Pien G.W., Ratcliffe S.J., Björnsdottir E., Arnardottir E.S., Pack A.I., Benediktsdottir B., Gislason T. (2014). The different clinical faces of obstructive sleep apnoea: A cluster analysis. Eur. Respir. J..

[17] Keenan B.T., Kim J., Singh B., Bittencourt L., Chen N.-H., Cistulli P.A., Magalang U.J., McArdle N., Mindel J.W., Benediktsdottir B. (2018). Recognizable clinical subtypes of obstructive sleep apnea across international sleep centers: A cluster analysis. Sleep.

[18] Messineo L., Bakker J.P., Cronin J., Yee J., White D.P. (2024). Obstructive sleep apnea and obesity: A review of epidemiology, pathophysiology and the effect of weight-loss treatments. Sleep Med. Rev..

[19] Von Elm E., Altman D.G., Egger M., Pocock S.J., Gøtzsche P.C., Vandenbroucke J.P. (2007). The Strengthening the Reporting of Observational Studies in Epidemiology (STROBE) statement: Guidelines for reporting observational studies. PLoS Med..

[20] Chiner E., Arriero J.M., Signes-Costa J., Marco J., Fuentes I. (1999). Validation of the Spanish version of the Epworth Sleepiness Scale in patients with a sleep apnea syndrome. Arch. Bronconeumol..

[21] Ng S.S.S., Chan T.-O., To K.-W., Ngai J., Tung A., Ko F.W.S., Hui D.S.C. (2010). Validation of Embletta portable diagnostic system for identifying patients with suspected obstructive sleep apnoea syndrome (OSAS). Respirology.

[22] Qaseem A., Dallas P., Owens D.K., Starkey M., Holty J.-E.C., Shekelle P. (2014). Diagnosis of obstructive sleep apnea in adults: A clinical practice guideline from the American College of Physicians. Ann. Intern. Med..

[23] Kapur V.K., Auckley D.H., Chowdhuri S., Kuhlmann D.C., Mehra R., Ramar K., Harrod C.G. (2017). Clinical Practice Guideline for Diagnostic Testing for Adult Obstructive Sleep Apnea: An American Academy of Sleep Medicine Clinical Practice Guideline. J. Clin. Sleep Med. JCSM Off. Publ. Am. Acad. Sleep Med..

[24] Sweatt K., Garvey W.T., Martins C. (2024). Strengths and Limitations of BMI in the Diagnosis of Obesity: What is the Path Forward?. Curr. Obes. Rep..

[25] Mazzotti D.R., Keenan B.T., Lim D.C., Gottlieb D.J., Kim J., Pack A.I. (2019). Symptom Subtypes of Obstructive Sleep Apnea Predict Incidence of Cardiovascular Outcomes. Am. J. Respir. Crit. Care Med..

[26] Nokes B., Orr J.E., White S., Luu S., Chen Z., Alex R., Sands S.A., Wojeck B.S., Owens R.L., Malhotra A. (2024). Effect of obesity on sleep apnea pathogenesis differs in women versus men: Multiple mediation analyses in the retrospective SNOOzzzE cohort. J. Appl. Physiol..

[27] Gottlieb D.J., Punjabi N.M. (2020). Diagnosis and Management of Obstructive Sleep Apnea: A Review. JAMA.

[28] Sutherland K., Lee R.W.W., Phillips C.L., Dungan G., Yee B.J., Magnussen J.S., Grunstein R.R., Cistulli P.A. (2011). Effect of weight loss on upper airway size and facial fat in men with obstructive sleep apnoea. Thorax.

[29] Ogilvie R.P., Patel S.R. (2017). The epidemiology of sleep and obesity. Sleep Health.

